# What are Juvenile-onset systemic sclerosis providers thoughts, experiences, and reasons for autologous stem cell transplant? Result of a multinational survey

**DOI:** 10.1177/23971983241293297

**Published:** 2024-11-08

**Authors:** Ivan Foeldvari, Samantha Branton, Suzanne C Li, Franziska J Rosser, Kathryn S Torok

**Affiliations:** 1 Hamburger Zentrum für Kinder-und Jugenrheumatologie, Hamburg, Germany; 2 Hamburg Centre for Pediatric and Adolescence Rheumatology, Centre for Treatment of Scleroderma and Uveitis in Childhood and Adolescence, Teaching Unit of the Asklepios Campus of the Semmelweis Medical School, Budapest, Schön Klinik Hamburg Eilbek, Hamburg, Germany; 3 UPMC Children’s Hospital of Pittsburgh, Pittsburgh, PA, USA; 4 Joseph M. Sanzari Children’s Hospital, Hackensack Meridian School of Medicine, Hackensack, NJ, USA; 5 Division of Pulmonary Medicine, Department of Pediatrics, School of Medicine, University of Pittsburgh, Pittsburgh, PA, USA; 6 Division of Rheumatology, Department of Pediatrics, School of Medicine, University of Pittsburgh, Pittsburgh, PA, USA

**Keywords:** Autologous bone marrow transplantation, CAR T cell therapy, cellular therapies, indication for transplantation, juvenile systemic sclerosis, juvenile systemic scleroderma

## Abstract

**Objective:**

Juvenile-onset systemic sclerosis (jSSc) is a rare and life-threatening disease with no formal studies evaluating the indications for, access to, or benefits of autologous stem cell transplantation (ASCT). As a first step toward understanding pediatric jSSc specialist thoughts and experiences with ASCT, we conducted a multinational survey.

**Methods:**

An electronic survey was developed and distributed in November 2023 to members of the Pediatric Rheumatology European Society (PRES) and/or Childhood Arthritis and Rheumatology Research Alliance (CARRA) pediatric scleroderma workgroups.

**Results:**

Twenty-nine (69%) jSSc specialists completed the survey. All participants have considered or would consider ASCT referral for a jSSc patient. Nearly all respondents indicated disease-modifying anti-rheumatic drugs (DMARDs) should be trialed prior to ASCT referral, with most indicating two to four DMARDs. The most common reasons selected for referral were rapidly progressive disease (despite DMARD) (90%), followed by severe disease status (83%), and significant impact on quality of life (83%). All respondents selected pulmonary disease as an indication for referral, followed by cardiac (93%), gastrointestinal (72%), and skin disease (66%). While pulmonary and cardiac involvement were considered individually sufficient for referral for ASCT, only a minority considered musculoskeletal involvement (28%) sufficient on its own.

**Conclusion:**

This survey is the first explore thoughts and experience with ASCT for jSSc. Results indicate pediatric rheumatologists were aware of and would consider ASCT for their patients. Our results indicate there is likely some variability in clinical practice regarding who is referred for ASCT, and further research is needed to guide development of evidence-based clinical care guidelines.

## Introduction

Juvenile-onset systemic sclerosis (jSSc) is a rare disease, affecting approximately 3 in 1,000,000 children.^1^ Similar to the adult-onset form, it causes the same life-threatening problems including interstitial lung disease and cardiac dysfunction, although generally has a lower mortality rate.^2,3^ Early initiation of currently available treatments, including biologic and non-biologic DMARDs, can slow or halt disease progression. However, progression toward end-stage organ damage still occurs in many patients despite these medication regimens, highlighting the need for additional treatment options.^4^

Hematopoietic Autologous Stem Cell Transplant (ASCT) has been shown to be a promising treatment option for many adults with SSc, with several controlled studies demonstrating its association with improved long-term survival.^5–7^ Currently, there are no clear guidelines regarding the indication of ASCT for jSSc patients, though two recent pediatric rheumatology consensus statements support its role in treatment-refractory progressive jSSc.^4,8^ The main issue for both adult and pediatric SSc patients is in identifying the “right patient” at the “right time”—those who do not sufficiently respond to conventional treatment and have fast disease progression and/or high disease burden,^9^ as transplant related mortality remains a concern for ASCT.

Given the paucity of existing data on jSSc specialists thoughts regarding access to-, indications for-, or optimal patient for ASCT, we developed and conducted a survey of pediatric jSSc experts.

## Methods

An electronic Qualtrics survey was developed through an iterative process with input from three pediatric rheumatologists (I.F., S.L., and K.T.) and one pediatric pulmonologist (F.R.), all with expertise in jSSc. The final survey contained up to 39 questions and utilized branching logic to improve applicability of questions and reduce survey fatigue (Supplementary Tables A-H). The survey was distributed via email to members of the, PRES Scleromderma working group members and the Childhood Arthritis and Rheumatology Research Alliance (CARRA) juvenile scleroderma working groups, as well as to the contributing physicians of the international inceptions cohort for patients with jSSc^10^ on November 8, 2023 with closure of survey on November 28, 2023. Survey data was captured anonymously. It was assured, if one person was present in one then more list, they were mailed only once.

The survey was divided into four themes: baseline characteristics of the respondent (n = 5 questions); familiarity with and access to ASCT (n = 3 questions); experience and consideration for jSSc-ASCT (n = 3 questions); indications for jSSc-ASCT and where indicated, six organ specific question sets (n = up to 28 questions, organ question sets 3–4 questions in length). An exploratory question about familiarity with Chimeric Antigen Receptor T-cell (CAR T-cell) therapy (n = 1) and an optional open-ended text question to provide comments was asked at the end of the survey.

Following survey data capture, three responses were identified as “spam” by Qualtrics and removed from analysis. Descriptive statistics for survey responses were obtained in Excel and were summarized.

This study did not require IRB approval.

## Results

The survey link was emailed as a Qualtrics link to 42 jSSc specialists, each from a different center, of whom 29 (69%) participated. Demographic information for those who did not complete the survey is unavailable. The average time to complete the survey was 9.59 min. Table 1 displays baseline characteristics. Respondents were from three continents: North America (52%), Europe (45%), and South America (3%). Almost all participants identified practicing in an academic center (79%), as a pediatric practice or healthcare system (97%), and their specialty as pediatric rheumatologist (97%). The number of jSSc patients followed in practice varied from 0 to > 20, with most respondents following between 1 and 10 (80%) (Table 1).

**Table 1.** Characteristics of respondents completing the survey and general information regarding ASCT for Juvenile-onset Systemic Sclerosis (jSSc).

Characteristics		N (%)
Number of jSSc patients in	0	1 (3%)
Practice	1–2	8 (28%)
	3–5	6 (21%)
	6–10	9 (31%)
	11–20	3 (10%)
	>20	2 (7%)
Practice type	Academic (e.g., University affiliated)	23 (79%)
	Non-Academic & Non-private (e.g., government)	6 (21%)
General location of practice	Europe	13 (45%)
	North America	15 (52%)
	South America	1 (3%)
Type of healthcare or practice	Pediatric practice	28 (97%)
	Adult practice	0 (0%)
	Both pediatric and adult practice	1 (3%)
Specialty	Pediatric rheumatologist	28 (97%)
	Other	1 (3%)
ASCT performed (any) in healthcare system or practice	Yes	23 (79%)
	No	6 (21%)
ASCT for systemic sclerosis within healthcare system or practice	No	13 (45%)
	jSSc only	4 (14%)
	aSSc only	1 (3%)
	Both jSSc and aSSc	11 (38%)
Care for any jSSc patients who have received an ASCT	Yes	11 (38%)
	No	18 (62%)
Ever considered ASCT for a jSSc patient	Yes	21 (72%)
	No	8 (28%)
Ever referred a jSSc patient for an ASCT	Yes	14 (48%)
	No, but I would refer if I had a patient that I thought would benefit or if there was an ASCT center I could refer patients to	15 (52%)
	No, I would NOT refer because I do not think ASCT is an appropriate therapeutic option at this time	0 (0%)

For all questions, n = 29 respondents. aSSc = Adult-onset Systemic Sclerosis. ASCT = Autologous Stem Cell Transplant.

Regarding ASCT, all respondents reported their practice or healthcare system performed ASCT for any condition or age, with fewer (55%) reporting their practice or healthcare performed ASCT for Systemic Sclerosis. Of those reporting their practice or healthcare system provided ASCT for SSc, 4 provided ASCT for jSSc only, 1 provided for adult-onset SSc (aSSc) only, and 11 provided for both jSSc and aSSc (Table 1). Although most respondents had not cared for a jSSc patient who had received an ASCT (62%), most reported having considered an ASCT for a jSSc patient (72%). Almost all participants had referred a jSSc patient for ASCT (48%) or would refer if they had a patient who would benefit or if they had an ASCT center to refer to (52%). No respondents selected the answer choice “no- I would not refer because I do not think ASCT is an appropriate therapeutic option at this time” (Table 1).

Regarding indications for ASCT referral, almost all respondents (90%) selected rapidly progressive jSSc disease despite a disease-modifying anti-rheumatic drug (DMARD) as an indication for ASCT referral, 83% selected severe impairment of quality of life and severe jSSc disease status, with fewer respondents selecting slowly progressive disease despite DMARD therapy (34%), age of jSSc patient (28%), or parental/caregiver preference (10%) (Table 2). Of the 23 respondents that ranked reasons for ASCT referral, rapidly progressive disease despite DMARD therapy was the most common reason selected (57%), followed by severe jSSc disease (35%). Of those that selected progressive disease despite DMARD (either slow or rapid), most selected a time interval for progression of 3–6 months (50%) or 6–12 months (42%) as the most important interval, with 4% selection 1–3 months or 12–24 months (Table 2). Almost all of the respondents (97%) thought DMARD should be tried prior to considering an ASCT, of which the majority of respondents (57%) indicated two to three DMARDs should be tried prior to ASCT, 21% indicated three to four DMARDS should be tried, and 11% indicated one^1^ DMARD and ⩾5 DMARDS should be tried (Table 2). Of the 28 respondents who indicated DMARDS should be tried prior to ASCT referral, the most common failed DMARDs to consider ASCT referral were mycophenolate (89%), cyclophosphamide (82%), tocilizumab (79%), methotrexate (68%), and rituximab (64%). Fewer respondents selected failure of abatacept (25%), JAK inhibitor (21%), or imatinib (4%) as a consideration for ASCT.

**Table 2.** Indications for autologous stem cell transplant for juvenile-onset systemic sclerosis.

Question	Answer choices	# respondents	N (%)
Reasons for ASCT referral (select all)	Slowly progressive despite DMARD	29	10 (34%)
	Rapidly progressive despite DMARD		26 (90%)
	Severe disease status		24 (83%)
	Age of patient		8 (28%)
	Parental/caregiver preference		3 (10%)
	Severe impairment of daily life		24 (83%)
Should DMARD(s) be tried first, prior to ASCT referral	Yes	29	28 (97%)
	No		1 (3%)
How many DMARD(s) should be tried prior to ASCT	1	28	3 (11%)
	2–3		16 (57%)
	3–4		6 (21%)
	⩾5		3 (11%)
Failure of which DMARD(s) would be a consideration	Methotrexate	28	19 (68%)
	Mycophenolate		25 (89%)
	Cyclophosphamide		23 (82%)
	Rituximab		18 (64%)
	Tocilizumab		22 (79%)
	Imatinab		1 (4%)
	Abatacept		7 (25%)
	JAK inhibitor		6 (21%)
	Other not listed		0 (0%)
Time interval most important for referral	1–3 months 3–6 months 6–12 months 12–24 months	26	1 (4%) 13 (50%) 11 (42%) 1 (4%)
Disease of which organ system would be consideration for ASCT referral (check all)	Skin	29	19 (66%)
	Pulmonary		29 (100%)
	Gastrointestinal		21 (72%)
	Musculoskeletal		8 (28%)
	Cardiac		27 (93%)
	Vascular		18 (62%)
Ranked most important (#1) organ system prompting a ASCT referral	Skin	29	1 (3%)
	Pulmonary		20 (69%)
	Gastrointestinal		0 (%)
	Musculoskeletal Cardiac		0 (%) 8 (28%)
	Vascular		0 (%)
Familiar with CAR-T as treatment SSc	Yes	29	13 (45%)
	No		16 (55%)

ASCT = autologous stem cell transplant, jSSc = juvenile-onset systemic sclerosis, DMARD = disease-modifying anti-rheumatic drug.

Regarding major organ system involvement, which would be a consideration for jSSc, 100% of respondents selected pulmonary, 93% cardiac, 72% gastrointestinal, 66% skin, 62% vascular, and 28% musculoskeletal. When asked to rank the importance of organ system with regards to ASCT referral, pulmonary was ranked the most frequently (69%), cardiac was ranked by 28% of respondents, and skin involvement was ranked by only 1 respondent (3%) (Table 2). Organ system-specific questions were only asked of participants who selected the organ system as a reason for referral (Supplementary Tables C-H).

Regarding the sufficiency of an isolated organ system involvement for referral for ASCT, variability was appreciated among responses, noting the number of responses varied by organ system (Supplementary Tables C-H). Most respondents indicated isolated pulmonary and cardiac disease would be sufficient (93% and 86%, respectively) for ASCT referral, whereas only around half of respondents indicated skin involvement, GI, and vascular involvement alone would be sufficient (i.e., additional organ system involvement would be needed).

**Figure 1.**
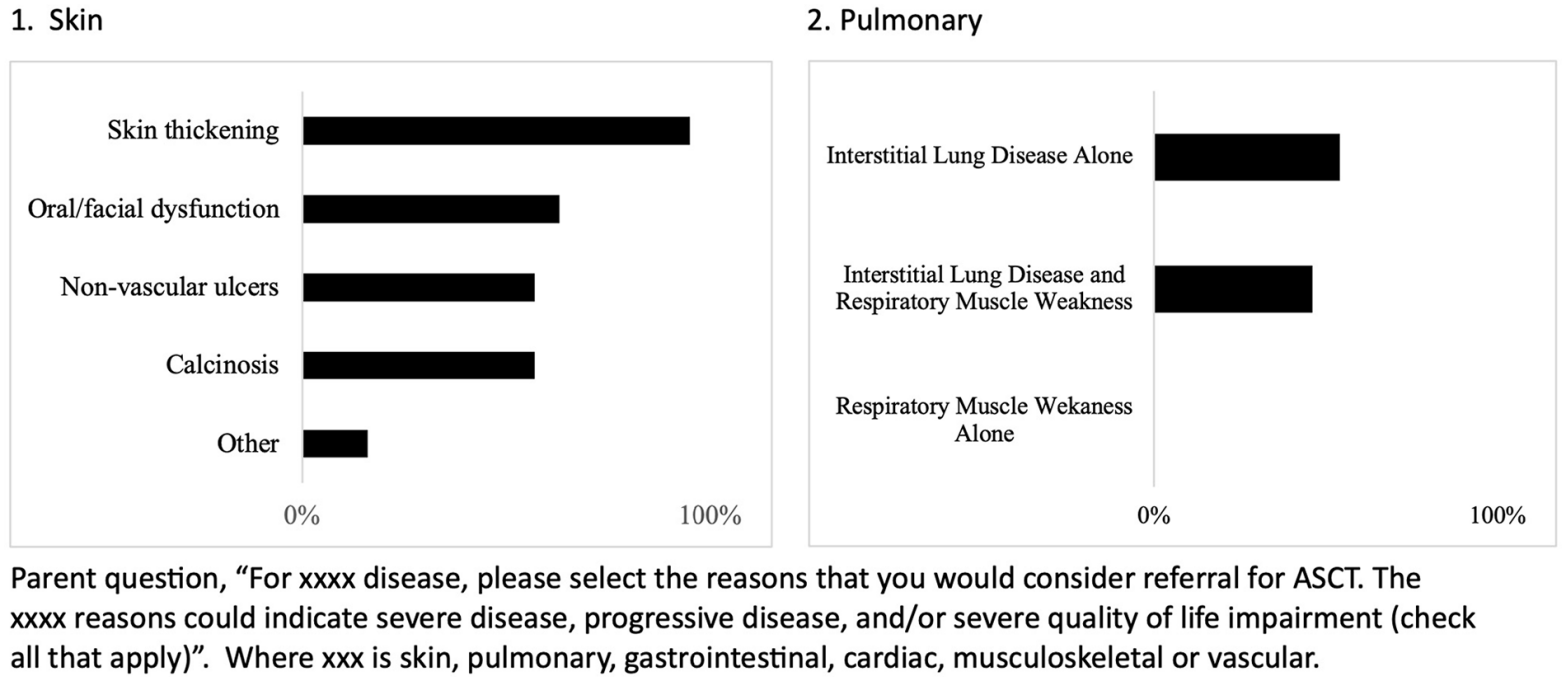
Indication for autologous stem cell transplant in juvenile systemic sclerosis within skin and pulmonary organ system.

**Figure 2.**
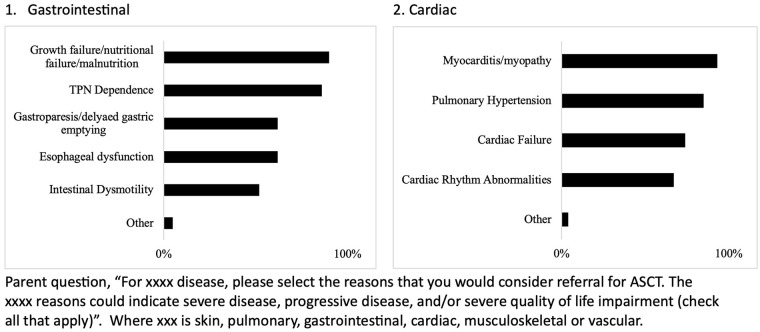
Indication for autologous stem cell transplant in juvenile systemic sclerosis within gastrointestinal and cardiac organ system.

**Figure 3.**
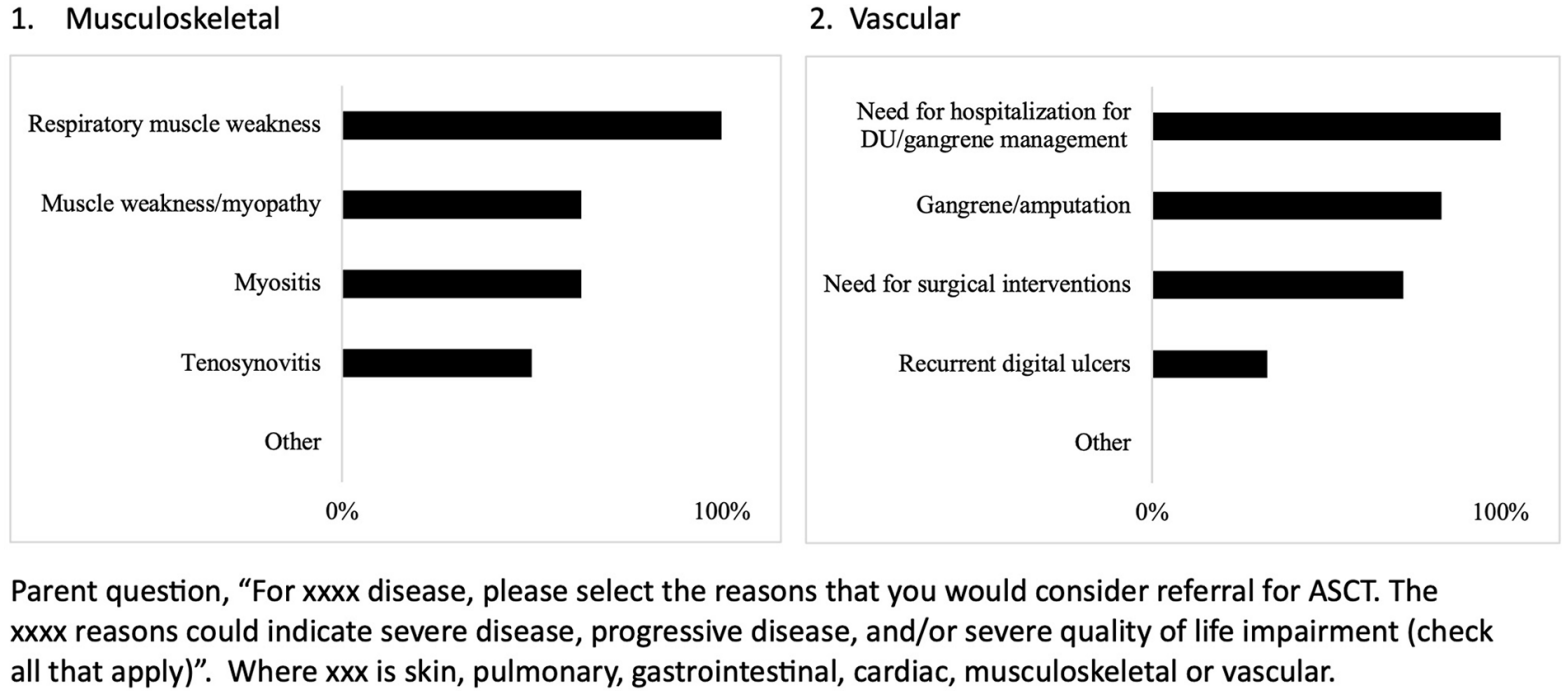
Indication for autologous stem cell transplant in juvenile systemic sclerosis within musculoskeletal and vascular organ system.

Indication responses per each of the six organ systems are summarized in Figures 1–3. Within organ systems, variation was appreciated regarding reasons for considering referral for ASCT (Supplementary Tables C-H). For example, for pulmonary involvement, half of respondents indicated interstitial lung disease (ILD) only as an indication (56%), whereas half of respondents indicated both ILD and respiratory muscle weakness as a reason (46%). More consistent results were found for skin, with skin thickening being identified by 95% of those who selected skin as an indication as a reason for ASCT and for GI, 90% selected growth failure/nutritional failure/malnutrition as an indication, and 100% of respondents who indicated vascular involvement as consideration for referral selected need for hospitalization for digital ulcers/gangrene management as a reason. Interestingly, pulmonary hypertension was selected as a reason by 85% of respondents indicating cardiac involvement as a reason for referral, whereas in adult ASCT clinical trials, pulmonary hypertension was an exclusion criterion. Fewer respondents indicated musculoskeletal involvement would be an indication for referral, and variation was appreciated for reasons other than respiratory muscle weakness (Supplementary Table G).

Indication for autologous stem cell transplant in juvenile systemic sclerosis within musculoskeletal and vascular organ system. When asked, most respondents indicated consideration of both the severity of disease and progression of disease, over progression alone for both skin and pulmonary involvement (Supplemental Tables C and D). For example, respondents indicated both low forced vital capacity (FVC) and progressive worsening of FVC more frequently as an indication (75%) compared to progressive worsening of FVC (21%) or low FVC (0%).

In an exploratory question regarding familiarity with Chimeric Antigen Receptor T-cell (CAR T) therapy, a potential novel treatment in adult SSc aimed at “resetting” the immune system, slightly over half of the respondents (55%) indicated familiarity (Table 2).

The final question of the survey was open-ended, inviting respondents to share any thoughts or comments about ASCT for jSSc. A summary of the key themes of these comments includes:

The need to consider disease activity versus disease damage when referring for ASCT.
The importance of assessing infectious risks associated with ASCT.
The need to weigh the severity of involvement of certain organ systems.
Difficult to know when to start the process of referral for ASCT.

## Discussion

Juvenile systemic sclerosis (jSSc) remains a life-threatening condition with a significant mortality rate compared to other pediatric rheumatologic conditions, particularly during the transition age (older teen and young adult).^11^ A US study underscored this risk, with similar findings reported in a cross-sectional international pediatric rheumatology survey.^2^ In this study, among 134 jSSc patients, 16 patients died, with four deaths within the first year of diagnosis and the remaining ten between the second and fifth year, resulting in a 5 year mortality of 12%.^2,4^

There is a clear need for more innovative treatments for certain jSSc patients, such as autologous stem cell transplant (ASCT), which offers the potential to reset the immune system. The SHARE guidelines recommend ASCT as a therapeutic option for progressive disease refractory to immuno-suppressive (DMARD) therapy,^8^ and the current international guidance derived through the Delphi process and consensus supports this recommendation.^4^ However, these guidelines lack specific definitions of “progressive disease” and do not specify the number of DMARDs that must be failed to classify a patient as “refractory to immunosuppressive therapy.” This is likely due to the paucity of high-quality evidence-based clinical data supporting optimal intervals and therapy algorithms. Consequently, much remains unknown about the perspectives and experiences of pediatric healthcare providers regarding ASCT in jSSc patients.

Our survey indicates that jSSc providers are familiar with ASCT as a treatment option, with all respondents having considered or be willing to consider ASCT referral. Nearly all agreed that DMARDs should be tried and failed prior to ASCT consideration, though the optimal number of DMARDs tried remains unclear. The majority of respondents selected a range of two to four DMARDs, with progression measured over 3–12 months. In addition, all providers agreed that pulmonary disease is a primary consideration for ASCT referral, with cardiac and gastrointestinal involvement being the second and third most common considerations, respectively. These organ systems are associated with poor prognosis in pediatric cohorts^2,12^ and increased mortality in adults.^13^ However, these survey results diverge somewhat from adult SSc ASCT studies, which traditionally exclude or restrict patients with pulmonary arterial hypertension or significant cardiac involvement ^5–7^ Furthermore, gastrointestinal involvement, while not typically a criterion for adult SSc ASCT trials, was ranked as a priority for ASCT referral in jSSc by our respondents.

Regarding the type, extent and severity of organ system involvement, our survey indicates variation among respondents. Our data suggest that pulmonary and cardiac involvement alone may justify ASCT referral, while additional organ system impairment may be necessary for skin, GI, musculoskeletal and vascular systems. There is variation in the perceived importance of different organ system manifestations. For example, two-thirds of respondents indicated skin thickening as the most important reason for ASCT referral, while others prioritized calcinosis or non-vascular ulcers. Across organ systems in jSSc, respondents chose both disease progression and severity of disease as indicators for ASCT referral, whereas adult ASCT in adult SSc is more strongly considered in progressive disease, not severity alone.^5–7^

The 3- to 12-month time period to assess progression of jSSc aligns with the treat-to-target concept, which was introduced as guidance for jSSc in a current publication.^4^ There is still a huge need to identify trajectories that predict which patients should be assessed for ASCT and which patient would respond to conventional treatment as demonstrated by Keret et al.^14^ and Gregory et al.,^15^ resulting in similar skin and lung clinical improvement with a better safety profile at 24 months in adults.

This survey reflects the current perspectives of pediatric rheumatologist treating jSSc patients regarding ASCT referral. More data on prognostic trajectories are needed to predict which patients will benefit the most from ASCT and related novel cellular therapies, such as CAR T, enabling earlier application and use as a rescue therapy.

## Supplemental Material

sj-pdf-1-jso-10.1177_23971983241293297 – Supplemental material for What are Juvenile-onset systemic sclerosis providers thoughts, experiences, and reasons for autologous stem cell transplant? Result of a multinational survey Supplemental material, sj-pdf-1-jso-10.1177_23971983241293297 for What are Juvenile-onset systemic sclerosis providers thoughts, experiences, and reasons for autologous stem cell transplant? Result of a multinational survey by Ivan Foeldvari, Samantha Branton, Suzanne C Li, Franziska J Rosser and Kathryn S Torok in Journal of Scleroderma and Related Disorders

sj-pdf-2-jso-10.1177_23971983241293297 – Supplemental material for What are Juvenile-onset systemic sclerosis providers thoughts, experiences, and reasons for autologous stem cell transplant? Result of a multinational survey Supplemental material, sj-pdf-2-jso-10.1177_23971983241293297 for What are Juvenile-onset systemic sclerosis providers thoughts, experiences, and reasons for autologous stem cell transplant? Result of a multinational survey by Ivan Foeldvari, Samantha Branton, Suzanne C Li, Franziska J Rosser and Kathryn S Torok in Journal of Scleroderma and Related Disorders

sj-pdf-3-jso-10.1177_23971983241293297 – Supplemental material for What are Juvenile-onset systemic sclerosis providers thoughts, experiences, and reasons for autologous stem cell transplant? Result of a multinational survey Supplemental material, sj-pdf-3-jso-10.1177_23971983241293297 for What are Juvenile-onset systemic sclerosis providers thoughts, experiences, and reasons for autologous stem cell transplant? Result of a multinational survey by Ivan Foeldvari, Samantha Branton, Suzanne C Li, Franziska J Rosser and Kathryn S Torok in Journal of Scleroderma and Related Disorders

sj-pdf-4-jso-10.1177_23971983241293297 – Supplemental material for What are Juvenile-onset systemic sclerosis providers thoughts, experiences, and reasons for autologous stem cell transplant? Result of a multinational survey Supplemental material, sj-pdf-4-jso-10.1177_23971983241293297 for What are Juvenile-onset systemic sclerosis providers thoughts, experiences, and reasons for autologous stem cell transplant? Result of a multinational survey by Ivan Foeldvari, Samantha Branton, Suzanne C Li, Franziska J Rosser and Kathryn S Torok in Journal of Scleroderma and Related Disorders

sj-pdf-5-jso-10.1177_23971983241293297 – Supplemental material for What are Juvenile-onset systemic sclerosis providers thoughts, experiences, and reasons for autologous stem cell transplant? Result of a multinational survey Supplemental material, sj-pdf-5-jso-10.1177_23971983241293297 for What are Juvenile-onset systemic sclerosis providers thoughts, experiences, and reasons for autologous stem cell transplant? Result of a multinational survey by Ivan Foeldvari, Samantha Branton, Suzanne C Li, Franziska J Rosser and Kathryn S Torok in Journal of Scleroderma and Related Disorders

sj-pdf-6-jso-10.1177_23971983241293297 – Supplemental material for What are Juvenile-onset systemic sclerosis providers thoughts, experiences, and reasons for autologous stem cell transplant? Result of a multinational survey Supplemental material, sj-pdf-6-jso-10.1177_23971983241293297 for What are Juvenile-onset systemic sclerosis providers thoughts, experiences, and reasons for autologous stem cell transplant? Result of a multinational survey by Ivan Foeldvari, Samantha Branton, Suzanne C Li, Franziska J Rosser and Kathryn S Torok in Journal of Scleroderma and Related Disorders

sj-pdf-7-jso-10.1177_23971983241293297 – Supplemental material for What are Juvenile-onset systemic sclerosis providers thoughts, experiences, and reasons for autologous stem cell transplant? Result of a multinational survey Supplemental material, sj-pdf-7-jso-10.1177_23971983241293297 for What are Juvenile-onset systemic sclerosis providers thoughts, experiences, and reasons for autologous stem cell transplant? Result of a multinational survey by Ivan Foeldvari, Samantha Branton, Suzanne C Li, Franziska J Rosser and Kathryn S Torok in Journal of Scleroderma and Related Disorders

sj-pdf-8-jso-10.1177_23971983241293297 – Supplemental material for What are Juvenile-onset systemic sclerosis providers thoughts, experiences, and reasons for autologous stem cell transplant? Result of a multinational survey Supplemental material, sj-pdf-8-jso-10.1177_23971983241293297 for What are Juvenile-onset systemic sclerosis providers thoughts, experiences, and reasons for autologous stem cell transplant? Result of a multinational survey by Ivan Foeldvari, Samantha Branton, Suzanne C Li, Franziska J Rosser and Kathryn S Torok in Journal of Scleroderma and Related Disorders
