# Supplementary material for: What are Juvenile-onset systemic sclerosis providers thoughts, experiences, and reasons for autologous stem cell transplant? Result of a multinational survey
Source: J Scleroderma Relat Disord. 2024 Nov 8;10(2):163–9. doi: 10.1177/23971983241293297 (PMC11559529; doi:10.1177/23971983241293297)
Supplement: sj-pdf-1-jso-10.1177_23971983241293297 – Supplemental material for What are Juvenile-onset systemic sclerosis providers thoughts, experiences, and reasons for autologous stem cell transplant? Result of a multinational survey [file sj-pdf-1-jso-10.1177_23971983241293297.pdf]

Supplementary Table A: Survey Questions 1-11 with answer choices

Question	Answer Choices
1. Do you agree to participate?	-Yes<br>-No
2. How many patients with JSSC do you follow in your current practice?	- 0<br>- 1-2<br>- 3-5<br>- 6-10<br>- 11-20<br>- >20
3. What best describes your practice type?	-Academic (e.g. University affiliated)<br>-Non-Academic & Non-private (e.g., government)<br>-Private Practice<br>-Other
4. Please select the general location of your practice?	-Africa<br>-Asia<br>-Australia<br>-Europe<br>-North America<br>-South America
5. What best describes your healthcare facility with regards to seeing pediatric patients?	-Pediatric practice<br>-Adult practice<br>-Both pediatric and adult practice<br>-I don't see any pediatric patients
6. What best describes your specialty?	-Pediatric Rheumatologist<br>- Other Pediatric Subspecialist<br>-Adult Rheumatologist<br>-Other Adult Subspecialist<br>-Other
7. Does your practice or healthcare system perform ASCT for any condition or age?	-Yes<br>-No
8. Does your healthcare system or practice provide ASCT for Systemic Sclerosis?	-No<br>-jSSc only<br>-adult SSc only<br>-both jSSc & adult-SSc
9. Do you provide care for any jSSc patients who have received an ASCT ? (the ASCT could have been done at another center)	-Yes<br>-No
10. Have you ever considered ASCT for a jSSc patient?	-Yes<br>-No
11. Have your ever referred a jSSc patient for an ASCT?	-Yes<br>-No, but I would refer if I had a patient that I thought would benefit or if there was an ASCT center I could refer patients to<br>-No, I would NOT refer because I do not think ASCT is an appropriate therapeutic option at this time
