# Supplementary material for: What are Juvenile-onset systemic sclerosis providers thoughts, experiences, and reasons for autologous stem cell transplant? Result of a multinational survey
Source: J Scleroderma Relat Disord. 2024 Nov 8;10(2):163–9. doi: 10.1177/23971983241293297 (PMC11559529; doi:10.1177/23971983241293297)
Supplement: sj-pdf-2-jso-10.1177_23971983241293297 – Supplemental material for What are Juvenile-onset systemic sclerosis providers thoughts, experiences, and reasons for autologous stem cell transplant? Result of a multinational survey [file sj-pdf-2-jso-10.1177_23971983241293297.pdf]

Supplementary Table B: Survey questions relating to reasons/indications for ASCT in jSSc

Question	Answer Choices
12. Please think about the reasons you would refer a JSSc patient for ASCT.<br>DMARD= disease modifying anti-rheumatic drugs. Check all that apply.<br><br>**only asked if has referred or would consider referring for ASCT (Q11) (N=29)*	-Slowly progressive jSSc (despite DMARD therapy)<br>-Rapidly progressive jSSc (despite DMARD therapy)<br>-Severe jSSc disease status<br>-Age of jSSc patient<br>-Parental/caregiver preference<br>-Severe impairment of quality of life
13. Please rank the importance of each indication for referral to ASCT (1=most important)<br><br>**only answer choices selected in question 12 available (N=23)*	-Slowly progressive jSSc (despite DMARD therapy)<br>-Rapidly progressive jSSc (despite DMARD therapy)<br>-Severe jSSc disease status<br>-Age of jSSc patient<br>-Parental/caregiver preference<br>-Severe impairment of quality of life
14. Do you think DMARD(s) should be tried first, prior to considering a referral for ASCT?<br>**only asked if has referred or consider referring for ASCT (Q11) (N=29)*	-Yes<br>-No
15. How many DMARD(s) should be tried prior to referring for ASCT?<br>**only asked if Q14 was yes (N=28)*	- 1<br>- 2-3<br>- 3-4<br>- 5 or more
16. Please review the list of DMARDS below. Failure of which DMARD(s) would be consideration for referring for ASCT? (check all that apply)<br><br>**only asked if Q14 was yes. (N=28)*	-Methotrexate<br>-Mycophenolate<br>-Cyclophosphamide<br>-Rituximab<br>-Tocilizumab<br>-Imatinab<br>-Abatacept<br>-JAK inhibitor<br>-Other not listed
17. For evidence of disease progression, what time interval is most important when considering referral for ASCT?<br>**only asked if Q13 indicated ASCT for slow or rapid progressive disease despite DMARD (N=26)*	- 1-3 months<br>- 3-6 months<br>- 6-12 months<br>- 12-24 months
18. Disease of which organ system(s) would be consideration for jSSc referral for ASCT? Check all that apply<br>**only asked if respondents answered yes to referring or considering referral for ASCT. (N=29)*	-Skin<br>-Pulmonary<br>-Gastrointestinal<br>-Musculoskeletal<br>-Cardiac<br>-Vascular

19. Rank the order of organ system involvement that would prompt a referral for ASCT in a jSSc patient.<br>1=most important<br>**only asked if respondents were asked question #18, and only organ systems selected in Q18 were displayed. (n=29)*	-Skin<br>-Pulmonary<br>-Gastrointestinal<br>-Musculoskeletal<br>-Cardiac<br>-Vascular
*denotes questions that utilized branching logic and may not have been asked of all respondents.	
