# Supplementary material for: What are Juvenile-onset systemic sclerosis providers thoughts, experiences, and reasons for autologous stem cell transplant? Result of a multinational survey
Source: J Scleroderma Relat Disord. 2024 Nov 8;10(2):163–9. doi: 10.1177/23971983241293297 (PMC11559529; doi:10.1177/23971983241293297)
Supplement: sj-pdf-3-jso-10.1177_23971983241293297 – Supplemental material for What are Juvenile-onset systemic sclerosis providers thoughts, experiences, and reasons for autologous stem cell transplant? Result of a multinational survey [file sj-pdf-3-jso-10.1177_23971983241293297.pdf]

Supplementary Table C: JSSc skin-specific organ involvement questions

Question	Answer	N (%)
20. For skin disease, choose the reasons that you would consider referral for ASCT. The skin reasons could indicate severe disease, progressive disease, and/or severe quality of life impairment (check all that apply) *(N=19)*	-Skin thickening<br>-Calcinosis<br>-Non-vascular ulcers<br>-Oral/facial dysfunction<br>-Other- not listed (list)	18 (95%)<br>11 (57%)<br>11 (57%)<br>12 (63%)<br>3 (16%)<br>(contractures)
21. For skin disease, please rank your reasons that you would consider referral for ASCT. The skin reasons could indicate severe disease, progressive disease, and/or severe quality of life impairment. *(N=13)*<br>**only answers selected in Q20 were available to rank*	 -Skin thickening<br>-Calcinosis<br>-Non-vascular ulcers<br>-Oral/facial dysfunction<br>-Other- not listed	**Ranked #1**<br>8 (62%)<br>2 (15%)<br>3 (23%)<br>0 (0%)<br>0 (0%)
22. For skin thickening as measured by modified Rodnan Skin Score (mRSS), which would you consider to be an indication for referral for ASCT? *(N=19)*	-High mRSS only<br>-Progressive worsening of mRSS only<br>-Both high or progressive worsening of mRSS<br>-None-mRSS is not important	0 (0%)<br>2 (11%)<br>17 (89%)<br><br>0 (%)
22. Would you refer for ASCT only because of skin disease severity or progressive worsening? *(N=19)*	-Yes<br>-No- would also need to have other organ system(s) with severe or worsening disease	10 (53%)<br>9 (47%)
These questions were only provided to the 19 respondents who selected skin as organ system involvement which would be a consideration for jSSc referral for ASCT (Question 18).		
