# Supplementary material for: What are Juvenile-onset systemic sclerosis providers thoughts, experiences, and reasons for autologous stem cell transplant? Result of a multinational survey
Source: J Scleroderma Relat Disord. 2024 Nov 8;10(2):163–9. doi: 10.1177/23971983241293297 (PMC11559529; doi:10.1177/23971983241293297)
Supplement: sj-pdf-4-jso-10.1177_23971983241293297 – Supplemental material for What are Juvenile-onset systemic sclerosis providers thoughts, experiences, and reasons for autologous stem cell transplant? Result of a multinational survey [file sj-pdf-4-jso-10.1177_23971983241293297.pdf]

Supplementary Table D: JSSc pulmonary-specific organ involvement questions

Question	Answer	N (%)
23. For pulmonary disease, please select your reasons that you would consider referral for ASCT. The pulmonary reasons could indicate severe disease, progressive disease, and/or severe quality of life impairment. *(N=28)*	- Interstitial lung disease only<br>-Respiratory muscle weakness only<br>-Both interstitial lung disease or respiratory muscle weakness<br>- Other- not listed	15 (54%)<br>0 (0%)<br>13 (46%)<br>0 (0%)
24. For pulmonary disease as measured by forced vital capacity (FVC), which would you consider to be an indication for referral to ASCT? *(N=28)*	-Low FVC only<br>-Progressive worsening of FVC only<br>-Both low FVC or progressive worsening of FVC<br>-None- FVC is not important or is not readily available in my area	0 (0%)<br>6 (21%)<br>21 (75%)<br>1 (4%)
25. For pulmonary disease as measured by chest CT scan, which would you consider to be an indication for referral to ASCT? *(N=29)*	-Extensive lung involvement on CT chest only<br>-Progressive worsening on CT chest only<br>-Both extensive involvement or progressive worsening on CT chest<br>-None- CT chest is not important or is not readily available in my area.	0 (0%)<br>4 (14%)<br>25 (86%)<br>0 (0%)
26. Would you refer for ASCT only because of lung disease severity, progressive worsening, or severe impairment of quality of life? *(N=29)*	Yes<br>-No- would also need to have other organ systems(s) with severe or worsening disease	27 (93%)<br>2 (7%)
These questions were provided to the 29 respondents who selected pulmonary as organ system involvement which would be a consideration for jSSc referral for ASCT (Question 18).		
