# Supplementary material for: What are Juvenile-onset systemic sclerosis providers thoughts, experiences, and reasons for autologous stem cell transplant? Result of a multinational survey
Source: J Scleroderma Relat Disord. 2024 Nov 8;10(2):163–9. doi: 10.1177/23971983241293297 (PMC11559529; doi:10.1177/23971983241293297)
Supplement: sj-pdf-5-jso-10.1177_23971983241293297 – Supplemental material for What are Juvenile-onset systemic sclerosis providers thoughts, experiences, and reasons for autologous stem cell transplant? Result of a multinational survey [file sj-pdf-5-jso-10.1177_23971983241293297.pdf]

**Supplementary Table E**: JSSc gastrointestinal specific organ involvement questions

| Question | Answer | N (%) |
|---|---|---|
| 27. For gastrointestinal disease, please choose the reasons that you would consider referral for ASCT. The GI reasons could indicate severe disease, progressive disease, and/or severe quality of life impairment. (check all that apply). *(N=21)* | - Esophageal dysfunction<br>- Gastroparesis/delayed gastric emptying<br>- Intestinal dysmotility<br>- Growth failure/nutritional failure/malnutrition<br>- Total parenteral nutrition (TPN) dependence<br>- Other- not listed | 13 (62%)<br>13 (62%)<br>11 (52%)<br>19 (90%)<br>18 (86%)<br>1 (5%) (Progressive decrease BMI) |
| 28. For gastrointestinal disease, please rank the reasons that you would consider referral for ASCT. (check all that apply). **only selected answers in Q27 were available for ranking. (N=16)* | <br>- Esophageal dysfunction<br>- Gastroparesis/delayed gastric emptying<br>- Intestinal dysmotility<br>- Growth failure/nutritional failure/malnutrition<br>- Total parenteral nutrition (TPM) dependence<br>-Other- not listed | **Ranked #1**<br>4 (25%)<br>0 (0%)<br>0 (0%)<br>6 (38%)<br>6 (38%)<br>0 (0%) |
| 29. Would you refer to ASCT only because of GI disease severity, progressive worsening, or severe impairment of quality of life? *(N=21)* | Yes<br>No | 11 (52%)<br>10 (48%) |
| These questions were only provided to the 21 respondents who selected gastrointestinal as organ system involvement which would be a consideration for jSSc referral for ASCT (Question 18). | | |
