# Supplementary material for: What are Juvenile-onset systemic sclerosis providers thoughts, experiences, and reasons for autologous stem cell transplant? Result of a multinational survey
Source: J Scleroderma Relat Disord. 2024 Nov 8;10(2):163–9. doi: 10.1177/23971983241293297 (PMC11559529; doi:10.1177/23971983241293297)
Supplement: sj-pdf-6-jso-10.1177_23971983241293297 – Supplemental material for What are Juvenile-onset systemic sclerosis providers thoughts, experiences, and reasons for autologous stem cell transplant? Result of a multinational survey [file sj-pdf-6-jso-10.1177_23971983241293297.pdf]

Supplementary Table F: JSSc cardiovascular-specific organ involvement questions

Question	Answer	N (%)
30. For cardiovascular disease, please chose the reasons that you would consider referral for ASCT. The cardiovascular reasons could indicate severe disease, progressive disease, and/or severe quality of life impairment. *(Check all that apply) (N=27)*	- Pulmonary Hypertension<br>- Cardiac rhythm abnormalities<br>- Myocarditis/myopathy<br>- Cardiac Failure<br>- Other- not listed	23 (85%)<br>18 (67%)<br>25 (93%)<br>20 (74%)<br>1 (4%) (-)
31. For cardiovascular disease, please rank the reasons that you would consider referral for ASCT. The cardiovascular reasons could indicate severe disease, progressive disease, and/or severe quality of life impairment. **only selected answers in Q30 were available for ranking. (N=18)*	- Pulmonary Hypertension<br>- Cardiac rhythm abnormalities<br>- Myocarditis/myopathy<br>- Cardiac Failure<br>- Other- not listed	5 (28%)<br>0 (0%)<br>6 (33%)<br>7 (39%)<br>0 (0%)
32. Would you refer to ASCT only because of cardiac disease severity, progressive worsening, or severe impairment of quality of life? *(N=27)*	- Yes<br>- No – would also need to have other organ system(s) with severe or worsening disease	24 (89%)<br>3 (11%)
These questions were only provided to the 27 respondents who selected cardiac as organ system involvement which would be a consideration for jSSc referral for ASCT (Question 18).		
