# Supplementary material for: What are Juvenile-onset systemic sclerosis providers thoughts, experiences, and reasons for autologous stem cell transplant? Result of a multinational survey
Source: J Scleroderma Relat Disord. 2024 Nov 8;10(2):163–9. doi: 10.1177/23971983241293297 (PMC11559529; doi:10.1177/23971983241293297)
Supplement: sj-pdf-7-jso-10.1177_23971983241293297 – Supplemental material for What are Juvenile-onset systemic sclerosis providers thoughts, experiences, and reasons for autologous stem cell transplant? Result of a multinational survey [file sj-pdf-7-jso-10.1177_23971983241293297.pdf]

Supplementary Table G: JSSc musculoskeletal specific organ involvement questions

Question	Answer	N (%)
33. For musculoskeletal (MSK) disease, please chose the reasons that you would consider referral for ASCT. The MSK reasons could indicate severe disease, progressive disease, and/or severe quality of life impairment. (Check all that apply) *(N=8)*	- Tenosynovitis<br>- Myositis<br>- Muscle weakness/myopathy<br>- Respiratory muscle weakness<br>- Other- not listed	4 (50%)<br>5 (63%)<br>5 (63%)<br>8 (100%)<br>0 (0%)
34. For musculoskeletal (MSK) disease, please rank the reasons that you would consider referral for ASCT.<br>The MSK reasons could indicate severe disease, progressive disease, and/or severe quality of life impairment. (Check all that apply)<br>**only selected answers in Q33 were available for ranking. (N=7)*	 - Tenosynovitis<br>- Myositis<br>- Muscle weakness/myopathy<br>- Respiratory muscle weakness<br>- Other- not listed	**Ranked #1**<br>0 (0%)<br>2 (29%)<br>1 (14%)<br>4 (57%)<br>0 (0%)
35. Would you refer to ASCT only because of MSK disease severity, progressive worsening, or severe impairment of quality of life? *(N=8)*	- Yes<br>- No – would also need to have other organ system(s) with severe or worsening disease	3 (38%)<br>5 (62%)
These questions were only provided to the 8 respondents who selected musculoskeletal as organ system involvement which would be a consideration for jSSc referral for ASCT (Question 18).		
