# Supplementary material for: What are Juvenile-onset systemic sclerosis providers thoughts, experiences, and reasons for autologous stem cell transplant? Result of a multinational survey
Source: J Scleroderma Relat Disord. 2024 Nov 8;10(2):163–9. doi: 10.1177/23971983241293297 (PMC11559529; doi:10.1177/23971983241293297)
Supplement: sj-pdf-8-jso-10.1177_23971983241293297 – Supplemental material for What are Juvenile-onset systemic sclerosis providers thoughts, experiences, and reasons for autologous stem cell transplant? Result of a multinational survey [file sj-pdf-8-jso-10.1177_23971983241293297.pdf]

Supplementary Table H: JSSc vascular specific organ involvement questions

Question	Answer	N (%)
36. For vascular disease, please chose the reasons that you would consider referral for ASCT. The vascular reasons could indicate severe disease, progressive disease, and/or severe quality of life impairment. (Check all that apply) *(N=18)*	- Recurrent digital ulcers (DU)<br>- Gangrene/ autoamputation<br>- Need for hospitalization(s) for DU/gangrene management<br>- Need for surgical intervention(s) (e.g. sympathectomy, digital block, etc).<br>- Other-not listed	6 (33%)<br>15 (83%)<br>18 (100%)<br>13 (72%)<br><br>0 (0%)
37. For vascular disease, please rank the reasons that you would consider referral for ASCT. The vascular reasons could indicate severe disease, progressive disease, and/or severe quality of life impairment. (Check all that apply) **only selected answers in Q36 were available for ranking. (N=11)*	 - Recurrent digital ulcers (DU)<br>- Gangrene/ autoamputation<br>- Need for hospitalization(s) for DU/gangrene management<br>- Need for surgical intervention(s) (e.g. sympathectomy, digital block, etc).<br>- Other-not listed	**Ranked #1**<br>2 (18%)<br>5 (45%)<br>3 (27%)<br><br>1 (9%)<br>0 (%)
38. Would you refer to ASCT only because of vascular disease severity, progressive worsening, or severe impairment of quality of life? *(N=18)*	-Yes<br>-No – would also need to have other organ system(s) with severe or worsening disease	11 (61%)<br>7 (39%)
These questions were only provided to the 18 respondents who selected vascular as organ system involvement which would be a consideration for jSSc referral for ASCT (Question 18).		
